# New Naphthalene Derivatives from the Bulbs of *Eleutherine americana* with Their Protective Effect on the Injury of HUVECs

**DOI:** 10.3390/molecules23092111

**Published:** 2018-08-22

**Authors:** De-Li Chen, Mei-Geng Hu, Yang-Yang Liu, Rong-Tao Li, Meng Yu, Xu-Dong Xu, Guo-Xu Ma

**Affiliations:** 1 Hainan Branch of Institute of Medicinal Plant Development, Chinese Academy of Medicinal Sciences & Peking Union Medical College (Hainan Provincial Key Laboratory of Resources Conservation and Development of Southern Medicine), Haikou 570311, China; chendeli9999@163.com (D.-L.C.); eadchris@163.com (Y.-Y.L.); lirt99@126.com (R.-T.L.); jokeryml@163.com (M.Y.); 2 Institute of Medicinal Plant Development, Chinese Academy of Medical Sciences & Peking Union Medical College, No. 151, Malianwa North Road, Haidian District, Beijing 100193, China; humeigeng@outlook.com (M.-G.H.); xdxu2012@163.com (X.-D.X.)

**Keywords:** *Eleutherine americana*, naphthalene derivatives, HUVECs

## Abstract

Five new naphthalene derivatives, named Eleutherols A–C (**1**–**3**) and Eleuthinones B–C (**4**,**5**), together with three known compounds were isolated from the bulbs of *Eleutherine americana*. Their structures were elucidated on the basis of spectroscopic analysis including HR-ESI-MS, 1D and 2D NMR techniques. These compounds exhibited a potent effect against the injury of human umbilical vein endothelial cell (HUVECs) induced by high concentrations of glucose in vitro.

## 1. Introduction

**Figure 1:**
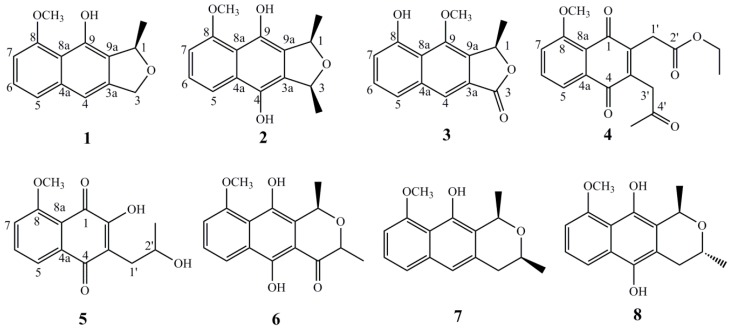
Structures of compounds **1**–**8**.

Hong-Cong (*Eleutherine americana* L. Merr.), a small plant that belongs to the Iridaceae family, is mainly distributed in South America, South Africa, and Southeast Asia [1,2]. The red bulbs of this plant (Hong-Cong in Chinese) have been long used as a folk medicine for the treatment of cardiac diseases, diabetes, breast cancer, stroke, hypertension, and sexual disorders, especially coronary disorder in the Hainan Island of South China [3,4,5,6]. Literature reported that the bulbs of Hong-Cong contained anthraquinones, naphthoquinones, and naphthalene derivatives and some ofthem displayed important biological activities, such as coronary vasodilating, prothrombin decreasing, antifertility, wound healing, topoisomerase II inhibitory, HIV inhibitory, antifungal, and anticancer activities [7,8,9,10,11,12]. As part of our ongoing investigation on the discovery of naturally occurring bioactive agents from medicinal plant, we examined the methanol extract of this plant and isolated five new naphthalene derivatives, named eleutherols A–C (**1**–**3**) and Eleuthinones B,C (**4**,**5**), together with three known compounds (**6**–**8**) (Figure 1). Herein, we report the isolation and structural elucidation of the isolatedones, as well as their protective effect on the injury of HUVECs (human umbilical vein endothelial cells) induced by high concentrations of glucose in vitro.

## 2. Results

**Figure 2:**
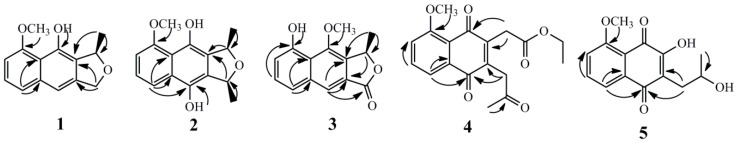
Key HMBC correlations of compounds **1**–**5**.

Compound **1** was obtained as yellow powder. Its molecular formula was deduced as C_14_H_14_O_3_ by the HR-ESI-MS data *m*/*z* 253.0824 [M + Na]^+^ (calcd. for 253.0841 C_14_H_14_NaO_3_). The UV spectrum showed absorption maxima at 220, 245, 265, and 420 nm. The IR spectrum exhibited the presence of hydroxyl group(s) at 3420 cm^−1^, aromatic ring(s) at 3060, 3015, 3005, 1615, 1578 cm^−1^ and ether linkage at 1240 and 1115 cm^−1^. In the ^1^H-NMR spectrum (Table 1) of compound **1**, one methyl signal at *δ*_H_ 1.61 (3H, d, *J* = 6.6 Hz), one methoxy signal at *δ*_H_ 4.06 (3H, s) and two mutual coupled oxygenated protons at *δ*_H_ 5.10 (1H, d, *J* = 12.6 Hz, Ha), 5.23 (1H, d, *J* = 12.6 Hz, Hb) were observed. The ^1^H-NMR spectrum also showed the resonances of three aromatic protons consistent with an ABM pattern at *δ*_H_ 6.75 (d, *J* = 7.8 Hz, H-7), 7.28 (t, *J* = 7.8 Hz, H-6) and 7.36 (d, *J* = 7.8 Hz, H-5). The ^13^C APT NMR spectrum (Table 1) of **1** exhibited 14 carbon signals including tenaromatic carbons (*δ*_C_ 103.7, 110.1, 114.7, 122.0, 125.2, 125.6, 137.5, 141.0, 148.4, and 156.8), two oxygenated carbons (*δ*_C_ 72.0, 79.5), one methoxy group at *δ*_C_ 56.3, one methylsignal at *δ*_C_ 20.6. The UV and IR patterns as well as the NMR data indicated compound **1** is a substituted naphthanolderivative which was further confirmed by 2D NMR spectra (See Supplementary) [13]. The connectivities of compound **1** were established mainly by HMBC correlations, shown in Figure 2. The-OCH_3_ group were assigned to C-8 judging from the downfield chemical shifts of C-8 (*δ*_C_ 156.8) and the HMBC correlations from the signal of *δ*_H_ 4.06 (3H, s, -OCH_3_) to C-8 (*δ*_C_ 156.8). The hydroxyl group was attached to C-9 on the basis of HMBC correlations from *δ*_H_ 9.44 to *δ*_C_ 148.4 together with the molecular formula C_14_H_14_O_3_ above. The HMBC correlations from the methyl protons at *δ*_H_ 1.61to C-1 (*δ*_C_ 79.5) and C-9a (*δ*_C_ 125.2) proved the presence of a methyl group located at C-1. In fact, the structure of **1** were similarly to the known compound Eleutherol [3,13], except for the absence of the carbonyl group at C-3. The absolute configuration of C-1 was established by ECD spectrum. In this experiment, the ECD spectrum (See Supplementary) of **1** showed a positive Cotton effect around 310 nm, suggested the configurationat C-1 to be *R* [14] [(2*S*) dihydroeleutherinol-8-*O*-*β*-d-glucopyranoside, CD *λ*_max_ 319 (−0.46)]. In addition, the optical rotation of **1** (${\left[\alpha \right]}_{D}^{20}$ = +12.5) also suggested a stereochemistry at C-1 to be *R* by comparing the optical rotations of (*S*) isoeleutherol (${\left[\alpha \right]}_{D}^{20}$ = −60.5) [13] and (*R*) (+)-Dihydroeleutherinol (${\left[\alpha \right]}_{D}^{25}$ = +8.8) [6]. Thus, the structure of **1** was elucidated as shown and named Eleutherol A.

Compound **2** was isolated as a yellow solid. HR-ESI-MS gave a quasi-molecular ion peak at *m*/*z* 283.0912 in the positive mode (Calcd. for 283.0946). Taking together with the analysis of ^1^H and ^13^C APT NMR spectra, the molecular formula of **2** was deduced as C_15_H_16_O_4_. The IR spectrum exhibited the presence of hydroxyl group(s) at 3415 cm^−1^, aromatic ring(s) at 3065, 3015, 3005, 1625, 1575 cm^−1^ and ether linkage at 1235 and 1105 cm^−1^. A detailed comparison of the NMR data between **2** and **1** revealed that there were one additional hydroxyl and methyl signals in **2**. These findings were fully supported by 2D NMR spectra (See Supplementary). In the HMBC spectrum, the correlations from *δ*_H_ 1.64 (3H, d, *J* = 6.6 Hz) to *δ*_C_ 67.3 (C-3), *δ*_H_ 12.83 (1H, s) to *δ*_C_ 154.4 (C-4) suggested the extra methyl and hydroxyl signals were attached to C-3 and C-4, respectively. The relative configuration of compound **2** was established by NOESY spectrum. In the experiment, the NOE enhancements from H-1 to H-3 indicated the synperiplanar position of CH_3_-1 and CH_3_-3. The similar ECD spectra between **2** and **1** exhibited the *R* configuration of C-1 in **2**. Therefore, compound **2** was determined as shown and named Eleutherol B.

Compound **3** was a brown amorphous powder. Its molecular formula of C_14_H_12_O_4_ was in agreement with its HR-ESI-MS mass spectrum [M + Na]^+^ *m*/*z* 267.0679 (calcd. for 267.0633, C_14_H_12_NaO_4_). Its UV absorptions at 225, 265, and 345 nm indicated the presence of benzene ring(s). The IR spectrum showed absorption bands of one or more hydroxyl groups at 3371 cm^−1^ and ester carbonyl functionality at 1738^−1^. The ^1^H-NMR spectrum (Table 1) of **3** displayed one methyl at *δ*_H_ 1.74 (d, *J* = 6.6 Hz), one methoxy at *δ*_H_ 4.12 (s), one hydroxyl at *δ*_H_ 9.67 (s) and the resonances of aromatic protons H-5, H-6 and H-7 as an ABM system at *δ*_H_ 7.60 (d, *J* = 7.8 Hz), 7.41 (t, *J* = 7.8 Hz) and 6.94 (d, *J* = 7.8 Hz), respectively. The ^1^H-NMR data seemed identical to those of compound **1**. However, the ^13^C-NMR spectrum of **3** displayed one more carbonyl group at *δ*_C_ 170.6, which indicated the methylene at C-3 in **1** was oxygenated and formed to carbonyl group in **3**. Further analysis its HSQC data (See Supplementary) displayed that *δ*_H_ 4.12 (3H, s, -OCH_3_) had direct correlation with *δ*_C_ 56.4, *δ*_H_ 7.60 (1H, d, *J* = 7.8 Hz, H-5) with *δ*_C_ 123.7, *δ*_H_ 7.41 (1H, t, *J* = 7.8 Hz, H-6) with *δ*_C_ 126.6, *δ*_H_ 6.94 (1H, d, *J* = 7.8 Hz, H-7) with *δ*_C_ 106.3 and *δ*_H_ 7.91 (1H, s, H-4) with *δ*_C_ 116.6 and the HMBC spectrum (See Supplementary) exhibited the correlations from *δ*_H_ 4.12 (3H, s, -OCH_3_) to *δ*_C_ 149.2 (C-9) and *δ*_H_ 9.67 (1H, s, -OH) to *δ*_C_ 156.6 (C-8), respectively. All the 2D NMR above suggested that the methoxygroup was located at C-9 and hydroxyl group at C-8. Taken together with the similar ECD spectrum (See Supplementary), the structure of compound **3** was established as depicted and named Eleutherol C.

Compound **4** was obtained as a yellow-brown solid. Its molecular formula of C_18_H_18_O_6_ was established on the basis of its mass spectrum, [M + Na]^+^ *m*/*z* 353.1014 (calcd. for 353.1001, C_18_H_18_NaO_6_). The^1^HNMR spectrum (Table 2) displayed an ABM aromatic pattern at *δ*_H_ 7.74 (dd, *J* = 8.4, 1.2 Hz, H-5), 7.67 (t, *J* = 8.4 Hz, H-6) and 7.29 (d, *J* = 8.4 Hz, H-7) together with ten downfield carbons (*δ*_C_ 182.8, 143.8, 140.4, 184.4, 140.0, 119.4, 135.0, 117.9, 159.8, and 119.8) indicated the presence of naphthaquninone moiety. The resonances of a methylene protons at *δ*_H_ 3.78 (2H, s) and methyl protons *δ*_H_ 2.30 (s) as well as three carbons (*δ*_C_ 41.8, 30.1, 203.1) suggested the existence of 3-one-propyl side chain. Furthermore, the resonances of a methylene protons at *δ*_H_ 3.62 (2H, s) and ethyl protons *δ*_H_ 1.26 (3H, t, *J* = 6.0), 4.14 (2H, m) as well as four carbons (*δ*_C_ 33.2, 169.5, 14.1, 61.4) displayed the presence of ethylethanoylunit. A methoxy group that resonated at *δ*_H_ 4.00 was placed at C-8 according to the HMBC experiment (See Supplementary) (Figure 2). The 3-one-propyl chain was proposed to be at the C-3 position due to the HMBC correlations of H-3′ to C-3 and C-4. The existence of anethylethanoyl sidechain (-CH_2_CO_2_CH_2_CH_3_) was located at the C-2 position according to the HMBC correlation of the methylene protons H-1′ to C-1 and C-2. Therefore, the structure **4** was assigned as shown and named as Eleuthinone B.

Compound **5** was isolated as an orange amorphous powder and was determined as C_14_H_14_O_5_ by the HR-ESI-MS data *m*/*z* 285.0732 [M + Na]^+^, (calcd. for 285.0739, C_14_H_14_NaO_5_). The strong absorption bands at 1655 and 1715 cm^−1^ in the IR spectrum and absorption maxima at 220, 245, 265, 270, and 405 nm in the UV spectrum suggested the presence of a *p*-quinone moiety [5,6]. An examination of the ^1^H and ^13^C APT NMR (Table 2) showed the structure of **5** to be similar to that of **4**. Further analysis of the NMR data (See Supplementary) (Figure 2) of **5** indicated that the 3-one-propyl and ethylethanoyl groups in **4** were replaced by 2-hydroxyl-propyl and hydroxyl group, respectively in **5**. The downfield chemical shift of C-2 (*δ*_C_ 154.8) and upfield chemical shift of C-2′ (*δ*_C_ 67.6) in **5** together with the molecular formula C_14_H_14_O_5_aboveconfirmed these differences. However, the absence of proper model compounds to use as references made the assignment of the absolute configuration at C-2′ unreliable. As a result, the structure of **5** was deduced as shown and named as Eleuthinone C.

The known compounds were identified as hongconin (**6**) [10], Karwinaphthol A (**7**) [15] and dihydroisoeleutherin (**8**) [16], by comparing their ^1^H and ^13^C-NMR data with the reported literature.

**Figure 3:**
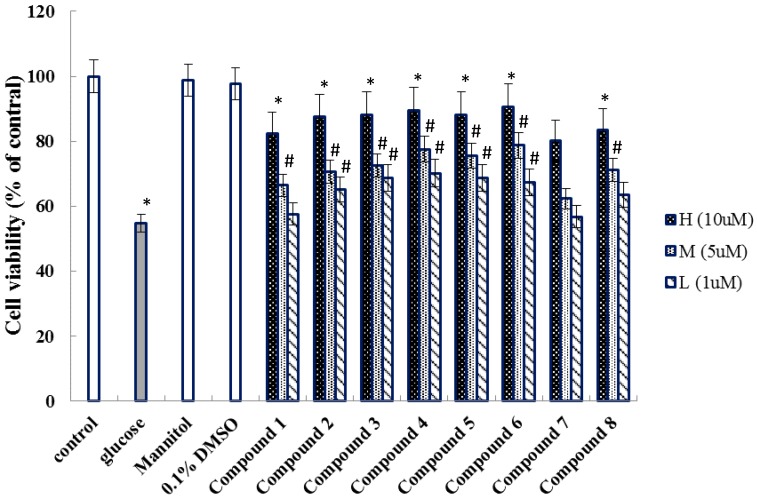
The protective effect of compounds **1**–**8** on high concentration glucose-induced viability of HUVECs. (mean ± SEM, *n* = 3); * *p* < 0.01 vs control; # *p* < 0.05 vs. Glucose. (Glucose: 30 mM; Mannitol: 30 mM).

Studies showed that the injury of HUVECs induced by high glucose was closely related to the cardiovascular disease [17,18]. Considering the civil applications of this medicinal herb, the isolated compounds **1**–**8** were studied for their protective effect on the injury of HUVECs induced by high glucose, in vitro (Figure 3).

## 3. Discussion

There are three main skeletons naphthalene, anthraquinone, and naphtoquinone, which have been isolated from *E. americana* [2].These structures, with the characteristics of a naphthalene ring and a furan ring or six-membered ring, were isolated from the red bulbs of *E. americana*, which are widely distributed in genus of *Eleutherine*. Ethnobotanically, the bulbs of plant are known for treating coronary abnormality. As a result, we investigated all the compounds for their protective effect on the injury of HUVECs activity. Comparing with the glucose group, all compounds displayed protective effect on the injury of HUVECs induced by high concentrations of glucose in vitro. Further analysis the data showed that compounds **2**–**6** exhibited better protective effect than other compounds, which indicated that the carbonyl group at furan or pyran ring in naphthalene skeleton may affect the pharmacological activity regarding HUVECs damage induced by glucose. With our knowledge, the evaluation of protective effect on the injury of HUVECs activity induced by glucose was the first time in vitro.

## 4. Materials and Methods

### 4.1. General Experimental Procedures

Optical rotations were obtained on a Perkin-Elmer 341 digital polarimeter (PerkinElmer, Norwalk, Waltham, MA, USA). UV and IR spectra were recorded on Shimadzu UV2550 and FTIR-8400S spectrometer (Shimadzu, Kyoto, Japan), respectively. ECD spectra were obtained using a JASCO J-815 spectro polarimeter. NMR spectra were obtained with a Bruker AV 600 NMR spectrometer (chemical shift are presented as *δ* values with TMS as the internal standard) (Bruker, Billerica, Germany). HR-ESI-MS were performed on a Q-tof spectrometer (Waters, Milford, MA, USA). Preparative HPLC was performed on an analytic LC equipped with a pump of P230, a DAD detector of 230+ (Ellte, Dalian, China), semi-preparative column. C18 ODS-A (50 μm, YMC, Kyoto, Japan) and Silica gel (100–200 and 200–300 mesh, Qingdao Marine Chemical Plant, Qingdao, China) was used for column chromatography. TLC analyses were carried out on Silica gel GF254 pre-coated plates (Zhi Fu Huang Wu Pilot Plant of Silica Gel Development, Yantai, China) with detection accomplished by spraying with 5% H_2_SO_4_ followed by heating at 100 °C. HUVECs cell was purchased at Shanghai cell bank of Chinese academy of sciences. All solvents used were of analytical grade (Beijing Chemical Works, Beijing, China).

### 4.2. Plant Material

The bulbs of *Eleutherine americana* L. Merr. were collected in November 2016 from Hechi, Guangxi Province and identified by Prof. Rong-Tao Li, Hainan Branch Institute of Medicinal Plant Development (Hainan Provincial Key Laboratory of Resources Conservation and Development of Southern Medicine), where the voucher specimens were conserved (No. 20161125GXEA). Plant drug was shade dried (<40 °C), coarsely powdered and stored in air tight container.

### 4.3. Extraction and Isolation

The dried and powdered bulbs of *E. americana* (5.0 kg) were extracted with methanol (50 L × 3) at room temperature for 24 h. The methanol extract was evaporated to dryness under reduced pressure to yield the extract (812.0 g). The residue was suspended to H_2_O (2.0 L) and partitioned with petroleum ether (3 × 2 L, MSO), CH_2_Cl_2_ (3 × 2 L), EtOAc (3 × 2 L) and n-BuOH (3 × 2 L), successively.

Fraction of the MSO (60.8 g) was subjected to column chromatography over silica gel (100–200 mesh) and eluted with MSO-CH_2_Cl_2_ (100:1) in increasing polarity. Then the fraction of MSO-CH_2_Cl_2_ (30:1) was subjected to column chromatography on silica gel and eluted in MSO-CH_2_Cl_2_ in gradient manner (from 80:1 to 0:100). Thin layer chromatography permitted to combine the resulted fractions which have the same *R*_f_ values into 6 series, Fr.A–F. Fr.C (0.75 g) was purified by HPLC with a gradient of 75% MeOH-H_2_O on an Agilent SB-Phenyl column to get compounds **1** (8.4 mg) in *R*_t_ 12.5 min, **2** (8.7 mg) in *R*_t_ 18.2 min, compound **7** (7.8 mg) in *R*_t_ 22.6 min and compound **8** (6.8 mg) in *R*_t_ 30.5 min. Fr.E (0.36 g) was separated by semi-preparative liquid chromatography using a MeOH-H_2_O (72:28) isocratic to yield **3** (9.6 mg, *R*_t_ 17.6 min), **6** (8.1 mg, *R*_t_ 23.4 min), **8** (10.2 mg, *R*_t_ 16.5 min) and **9** (8.6 mg, *R*_t_ 20.8 min). The entire detection was under UV 254 nm and the flow rate was 2 mL/min.

The structures of compounds **1**–**8** were determined by UV, IR, ^1^H-NMR, ^13^C-NMR , ^1^H-^1^H COSY, HSQC, HMBC, NOESY and HR-ESI-MS.

*Eleutherol A* (**1**). C_14_H_14_O_3_, yellow powder; ${\left[\alpha \right]}_{D}^{20}$ + 12.5 (*c* 0.1, MeOH); UV *λ*_max_ (CHCl_3_) nm (log *ε*): 220, 245, 265, 420; IR (KBr) *ν*_max_ cm^−1^: 1115, 1240, 1578, 1615, 3005, 3015, 3060, 3420; CD (MeOH, Δ*ε*) *λ*_max_ 310 (+0.24); HR-ESI-MS *m*/*z* 253.0824 [M + Na]^+^ (calcd. 253.0841); ^1^H and ^13^C-NMR spectra data, see Table 1.

*Eleutherol B* (**2**). C_15_H_16_O_4_, yellow brown solid; ${\left[\alpha \right]}_{D}^{20}$ + 36.7 (*c* 0.1, MeOH); UV *λ*_max_ (CHCl_3_) nm (log *ε*): 225, 240, 265, 275, 415; IR (KBr) *ν*_max_ cm^−1^: 1105, 1235, 1575, 1625, 3005, 3015, 3065, 3415; CD (MeOH, Δ*ε*) *λ*_max_ 325 (+0.22); HR-ESI-MS *m*/*z* 283.0912 [M + Na]^+^ (calcd. 283.0946); ^1^H and ^13^C-NMR spectra data, see Table 1.

*Eleutherol C* (**3**). C_14_H_12_O_4_, brown amorphous; ${\left[\alpha \right]}_{D}^{20}$ + 24.8 (*c* 0.1, MeOH); UV *λ*_max_ (CHCl_3_) nm (log *ε*): 225, 265, 345; IR (KBr) *ν*_max_ cm^−1^: 1108, 1235, 1575, 1738, 3015, 3371, 3680; CD (MeOH, Δ*ε*) *λ*_max_ 310 (+0.64), 255 (+2.25); HR-ESI-MS *m*/*z* 267.0679 [M + Na]^+^ (calcd. 267.0633); ^1^H and ^13^C-NMR spectra data, see Table 1.

*Eleuthinone B* (**4**). C_16_H_14_O_4_, yellow brown solid; UV *λ*_max_ (CHCl_3_) nm (log *ε*): 228, 243, 268, 272 and 405; IR (KBr) *ν*_max_ cm^−1^: 1355, 1458, 1585, 1635, 1735, 2848, 2940; HR-ESI-MS *m*/*z* 353.1014 [M + Na]^+^ (calcd. 353.1001); ^1^H and ^13^C-NMR spectra data, see Table 2.

*Eleuthinone C* (**5**). C_14_H_14_O_5_, orange amorphous powder; ${\left[\alpha \right]}_{D}^{20}$ − 6.7 (*c* 0.1, MeOH); UV *λ*_max_ (CHCl_3_) nm (log *ε*): 224, 248, 268, 272 and 405; IR (KBr) *ν*_max_ cm^−1^: 1355, 1466, 1585, 1605, 1720, 2845, 2940, 3320, 3630; HR-ESI-MS *m*/*z* 285.0732 [M + Na]^+^ (calcd. 285.0739); ^1^H and ^13^C-NMR spectra data, see Table 2.

### 4.4. Activity Assay

Compounds **1**–**8** were screened protective effect against the injury of HUVECs induced by high concentrations of glucose using the MTT method as described in the previous published literature [19] with appropriate modifications. Briefly, the cells were cultured in DMEM medium with 10% fetal bovine serum at 37 °C in a 5% CO_2_ incubator for 24 h. 100 μL of adherent cells was seeded on 96-well microtiter plate and allowed to adhere for 12 h. Then, the cells were treated with the test compounds at various concentrations (1 μM, 5 μM and 10 μM) for 2 h in triplicate. After 2 h of treatment, with the final concentration of 30 mM glucose was added directly into all the appropriate wells for 24 h. Absorbance were measured by the absorbance at 490 nm using a multiwell spectrophotometer.

## Figures and Tables

**Table 1:** ^1^H (600 MHz) and ^13^C-NMR (150 MHz) assignments of compounds **1**–**3** (CDCl_3_).

No.	1		2		3	
	*δ*_C_	*δ*_H_ (*J* in Hz)	*δ*_C_	*δ*_H_ (*J* in Hz)	*δ*_C_	*δ*_H_ (*J* in Hz)
1	79.5	5.53, q, 6.0	69.6	4.71, q, 6.6	78.1	5.74, q, 6.0
3	72.0	5.10, d, 12.6 5.23, d, 12.6	67.3	5.50, q, 6.6	170.6	
3a	141.0		120.9		127.9	
4	110.1	7.12, s	154.4		116.6	7.91, s
4a	137.5		126.0		137.2	
5	122.0	7.36, d, 7.8	118.1	8.04, d, 8.4	123.7	7.60, d, 7.8
6	125.6	7.28, t, 7.8	125.3	7.39, t, 8.4	126.6	7.41, t, 7.8
7	103.7	6.75, d, 7.8	109.1	7.02, d, 8.4	106.3	6.94, d, 7.8
8	156.8		155.7		156.6	
8a	114.7		107.7		117.5	
9	148.4		139.4		149.2	
9a	125.2		119.6		125.9	
CH_3_-1	20.6	1.61, d, 6.6	16.1	1.53, d, 6.6	19.2	1.74, d, 6.6
CH_3_-3			17.4	1.64, d, 6.6		
OCH_3_-8	56.3	4.06, s	56.2	4.07, s		
OCH_3_-9					56.4	4.12, s
4-OH				8.99, s		
8-OH						9.67, s
9-OH		9.44, s		12.83, s		

**Table 2:** ^1^H (600 MHz) and ^13^C-NMR (150 MHz) assignments of compounds **4**,**5** (CDCl_3_).

No.	4		5	
	*δ*_C_	*δ*_H_ (*J* in Hz)	*δ*_C_	*δ*_H_ (*J* in Hz)
1	182.8		179.6	
2	143.8		154.8	
3	140.4		118.7	
4	184.4		185.7	
4a	140.0		135.0	
5	119.4	7.74, dd, 8.4, 1.2	119.6	7.80, d, 8.4
6	135.0	7.67, t, 8.4	136.2	7.72, t, 8.4
7	117.9	7.29, d, 8.4	117.0	7.27, d, 8.4
8	159.8		160.2	
8a	119.8		116.8	
1′	33.2	3.62, s	33.0	2.81, dd, 13.2, 3.6 2.76, dd, 13.2, 7.8
2′	169.5		67.6	4.12, m
3′	41.8	3.78, s		
4′	203.1			
2′-OCH_2_CH_3_	61.4	4.14, m		
2′-OCH_2_CH_3_	14.1	1.26, t, 6.0		
2′-CH_3_			23.6	1.26, d, 6.6
4′-CH_3_	30.1	2.30, s		
8-OCH_3_	56.5	4.00, s	56.7	4.04, s

## Supplementary Materials

Supplementary Materials are available online.

## Abbreviations

The following abbreviations are used in this manuscript:

UV	Ultra violet
IR	Infrared
NMR	Nuclear magnetic resonance
HR-ESIMS	High resolution electrospray ionization mass spectroscopy
HMBC	Heteronuclear multiple bond correlation
HSQC	Heteronuclear single quantum correlation
COSY	Homonuclear chemical shift Correlation Spectroscopy
NOESY	Nuclear overhauser effect spectroscopy
ODS	Octadecylsilane
HPLC	High performance liquid chromatography
MSO	Petroleum ether
CH_2_Cl_2_	Dichloromethane
EtOAc	Ethyl acetate
nBuoH	n-Butanol
MeOH	Methanol
HUVECs	human umbilical vein endothelial cells
MTT	3-(4,5-Dimethylthiazol-2-yl)-2,5-diphenyltetrazolium bromide
DMEM	DuIbecco’s modified eagIe’s medium
