# Supplementary material for: New Naphthalene Derivatives from the Bulbs of Eleutherine americana with Their Protective Effect on the Injury of HUVECs
Source: Molecules. 2018 Aug 22;23(9):2111. doi: 10.3390/molecules23092111 (PMC6225190; doi:10.3390/molecules23092111)
Supplement: Supplementary file 1 [file molecules-23-02111-s001.pdf]

New Naphthalene Derivatives from the Bulbs of *Eleutherine americana* with their protective effect on the injury of HUVECs

De-Li Chen [1†], Mei-Geng Hu [2†], Yang-Yang Liu [1], Rong-Tao Li [1], Meng Yu [1,2], Xu-Dong Xu [2], Guo-Xu Ma [1, 2,*]

[1] Hainan Branch of Institute of Medicinal Plant Development, Chinese Academy of Medicinal Sciences & Peking Union Medical College (Hainan Provincial Key Laboratory of Resources Conservation and Development of Southern Medicine), Haikou 570311, China; chendeli9999@163.com (D.-L.C.); eadchris@163.com (Y.-Y.L.); lirt99@126.com (R.-T.L.); 18789087155@163.com (M.Y.)

[2] Institute of Medicinal Plant Development, Chinese Academy of Medical Sciences & Peking Union Medical College, No. 151, Malianwa North Road, Haidian District, Beijing 100193, China; humeigeng@outlook.com (M.-G.H.); xdxu2012@163.com (X.-D.X.)

* Correspondence: mgxfl8785@163.com; Tel.: +86-010-5783-3296

† Contributed equally to this work.

Received: 25 July 2018; Accepted: 19 August 2018; Published: date

Abstract: Five new naphthalene derivatives, named Eleutherols A-C (**1**-**3**) and eleuthinones B-C (**4**-**5**),, together with three known compounds were isolated from the bulbs of *Eleutherine americana*. Their structures were elucidated on the basis of spectroscopic analysis including HR-ESI-MS, 1D and 2D NMR techniques. These compounds exhibited a potent effect against the injury of human umbilical vein endothelial cell (HUVECs) induced by high concentrations of glucose in vitro.

Keywords: *Eleutherine americana*; Naphthalene derivatives; HUVECs

List of Figures S1-S26

Figure S1. ^{1}H-NMR (600 MHz, $CDCl_3$) spectrum of the new compound **1**

Figure S2. ^{13}C-APT (150 MHz, $CDCl_3$) spectrum of the new compound **1**

Figure S3. HSQC spectrum of the new compound **1**

Figure S4. HMBC spectrum of the new compound **1**

Figure S5. ^{1}H-NMR (600 MHz, $CDCl_3$) spectrum of the new compound **2**

Figure S6. ^{13}C-APT (150 MHz, $CDCl_3$) spectrum of the new compound **2**

Figure S7. HSQC spectrum of the new compound **2**

Figure S8. HMBC spectrum of the new compound **2**

Figure S9. NOESY spectrum of the new compound **2**

Figure S10. ^{1}H-NMR (600 MHz, $CDCl_3$) spectrum of the new compound **3**

Figure S11. ^{13}C-APT (150 MHz, $CDCl_3$) spectrum of the new compound **3**

Figure S12. HSQC spectrum of the new compound **3**

Figure S13. HMBC spectrum of the new compound **3**

Figure S14. ^{1}H-NMR (600 MHz, $CDCl_3$) spectrum of the new compound **4**

Figure S15. ^{13}C-APT (150 MHz, $CDCl_3$) spectrum of the new compound **4**

Figure S16. HSQC spectrum of the new compound **4**

Figure S17. HMBC spectrum of the new compound **4**

Figure S18. ^{1}H-NMR (600 MHz, $CDCl_3$) spectrum of the new compound **5**

Figure S19. ^{13}C-APT (150 MHz, $CDCl_3$) spectrum of the new compound **5**

Figure S20. HSQC spectrum of the new compound **5**

Figure S21. HMBC spectrum of the new compound **5**

Figure S22. ^{1}H-^{1}H COSY spectrum of the new compound **5**

Figure S23. NOESY spectrum of the new compound **5**

Figure S24. ECD spectrum of the new compound **1** in MeOH

Figure S25. ECD spectrum of the new compound **2** in MeOH

Figure S26. ECD spectrum of the new compound **3** in MeOH

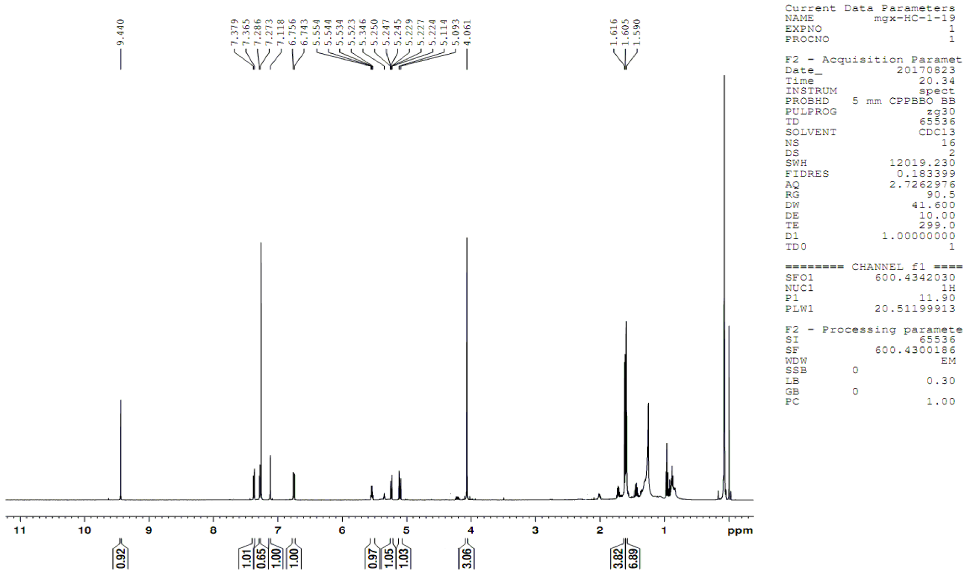

Figure S1. ^{1}H-NMR (600 MHz, $CDCl_3$) spectrum of the new compound **1**

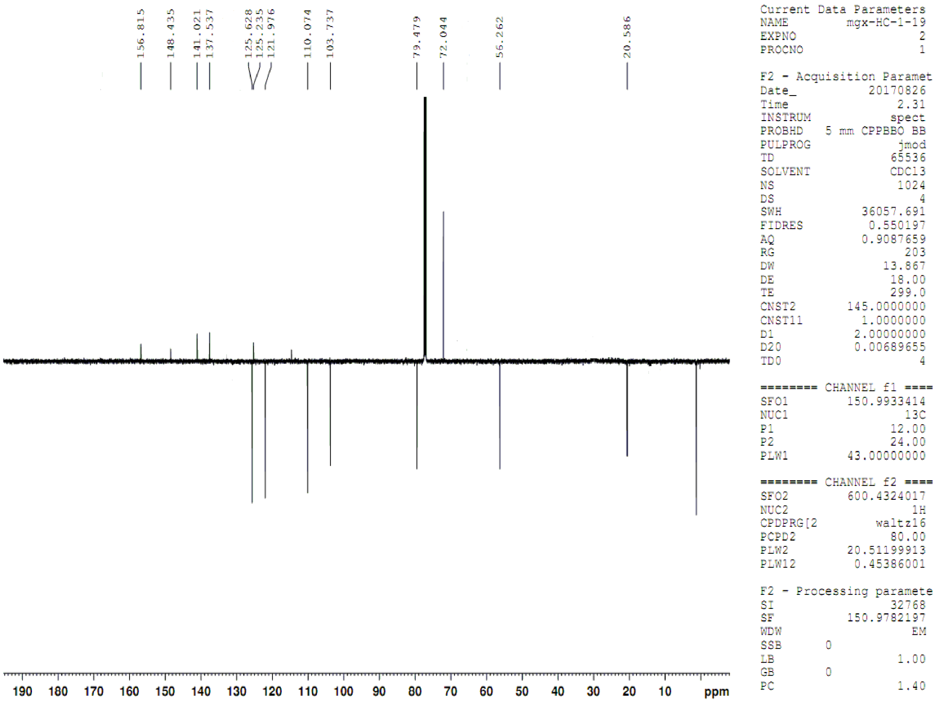

Figure S2. ^{13}C-APT (150 MHz, $CDCl_3$) spectrum of the new compound **1**

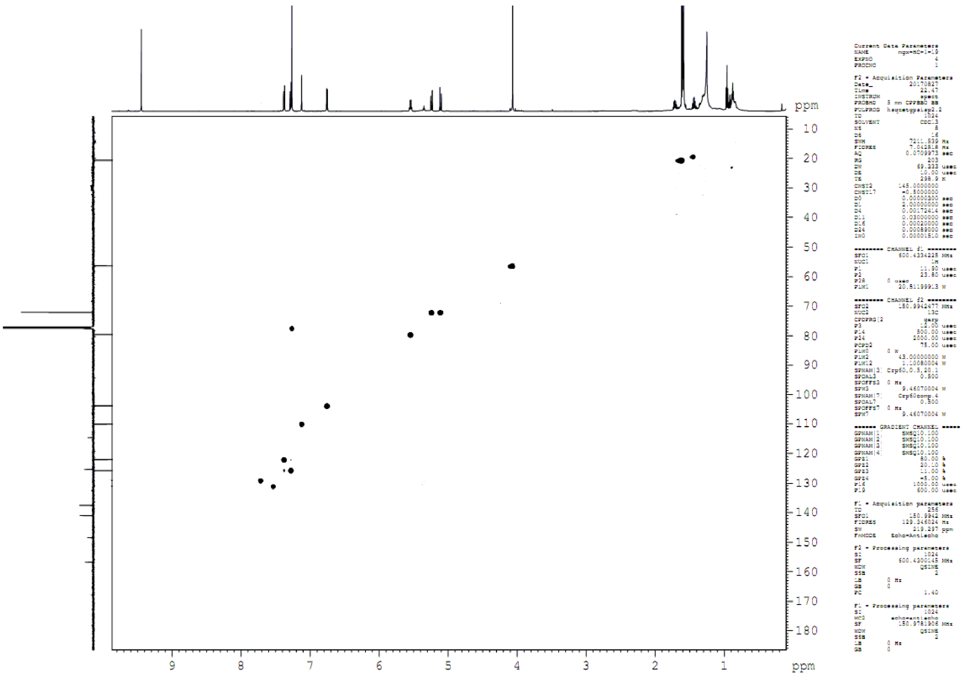

Figure S3. HSQC spectrum of the new compound **1**

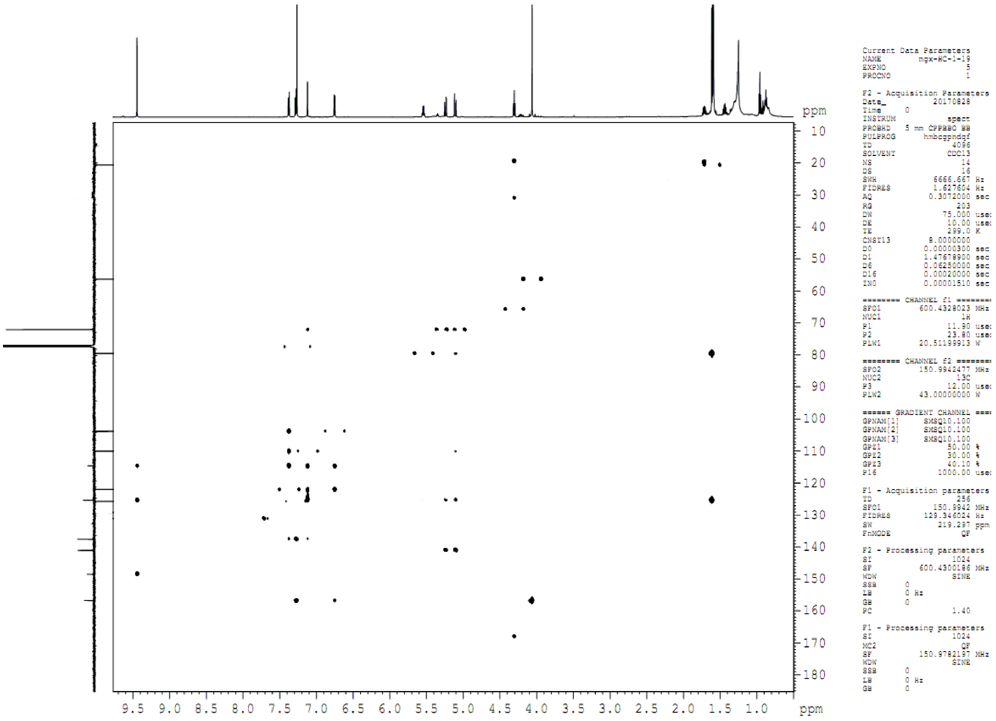

Figure S4. HMBC spectrum of the new compound **1**

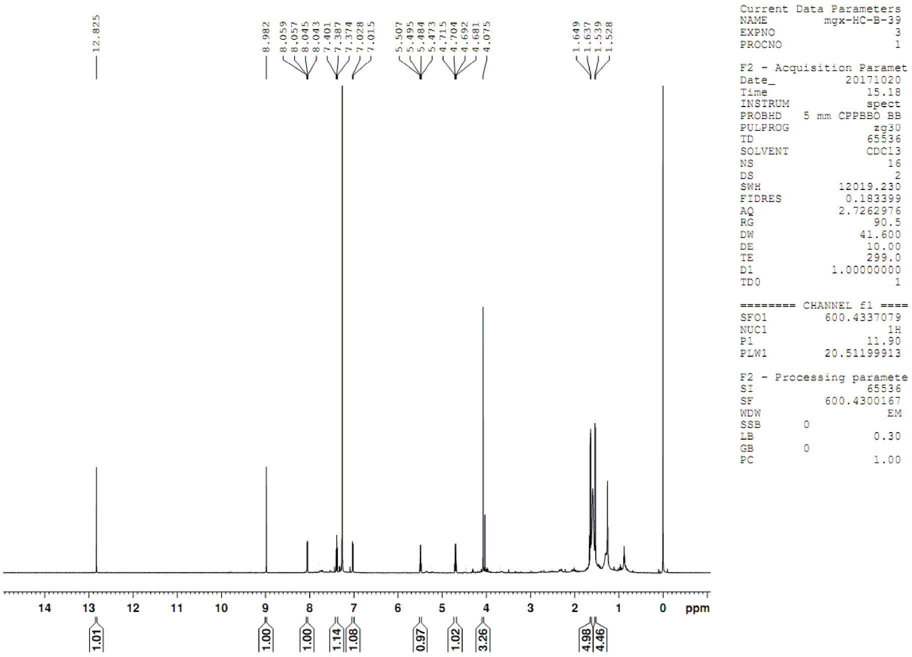

Figure S5. ^{1}H-NMR (600 MHz, $CDCl_3$) spectrum of the new compound **2**

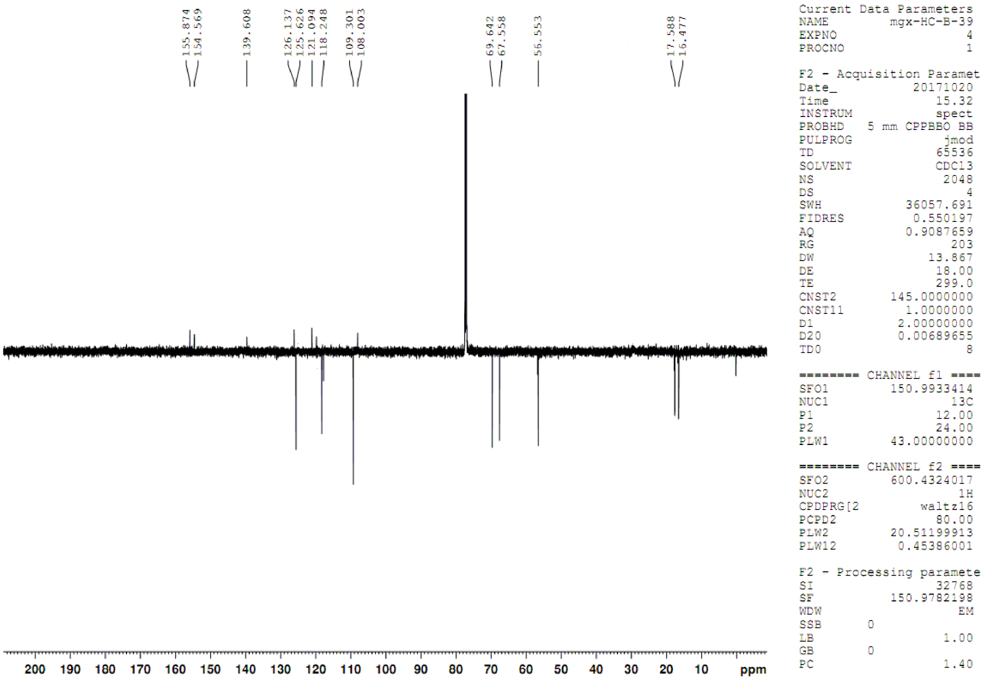

Figure S6. ^{13}C-APT (150 MHz, $CDCl_3$) spectrum of the new compound **2**

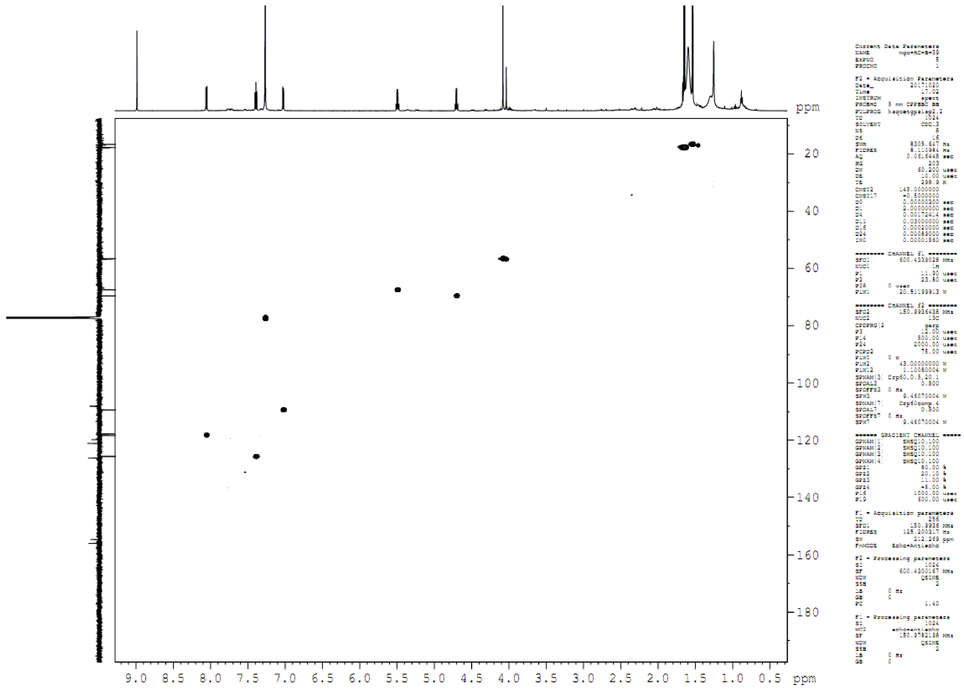

Figure S7. HSQC spectrum of the new compound **2**

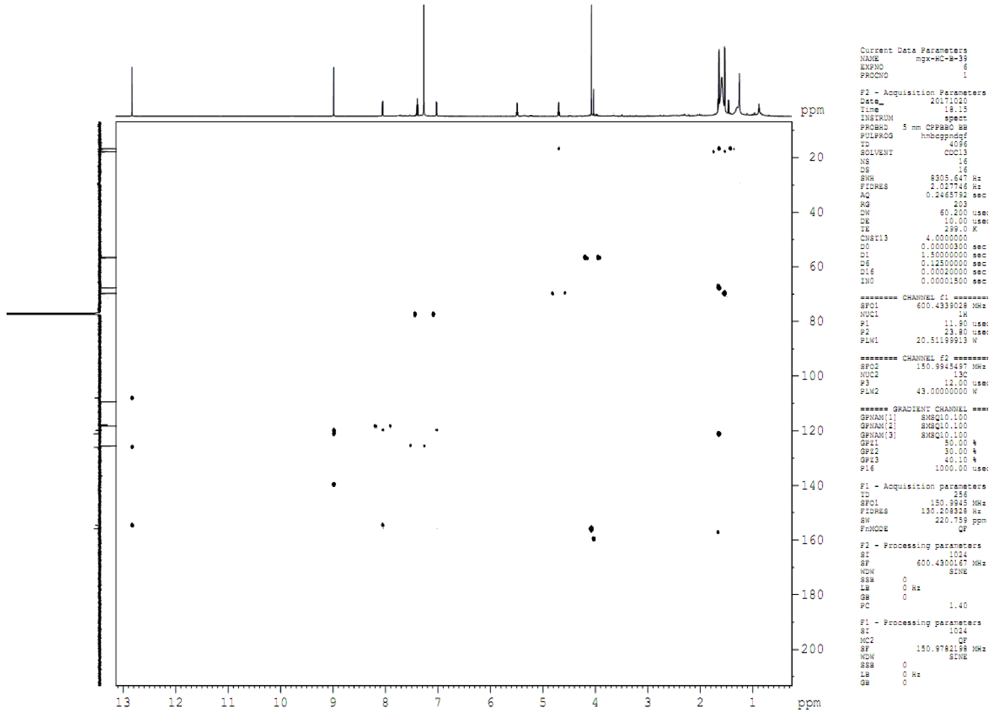

Figure S8. HMBC spectrum of the new compound **2**

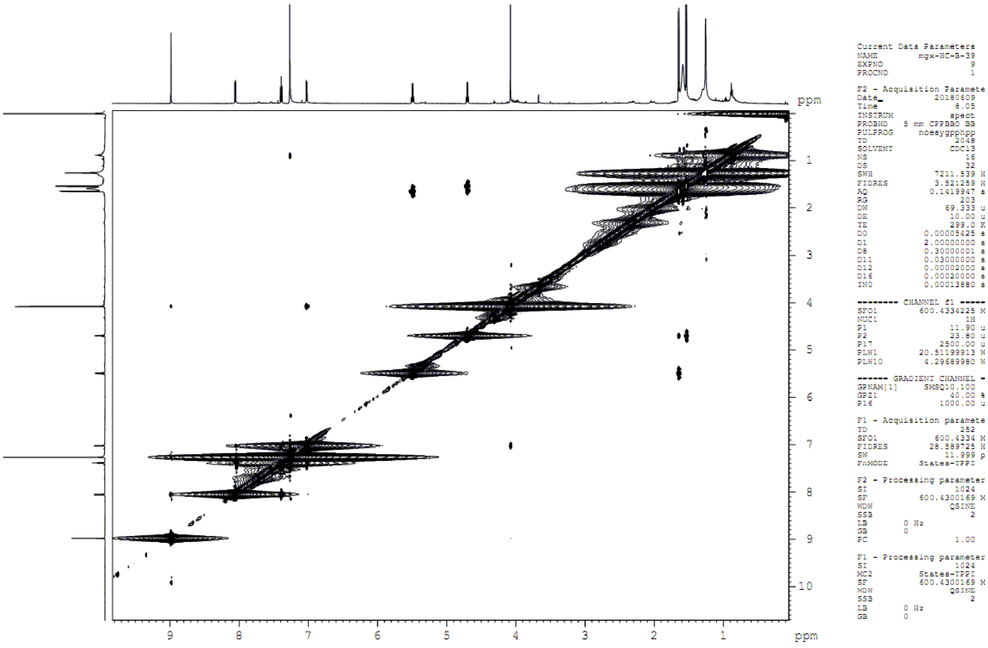

Figure S9. NOESY spectrum of the new compound **2**

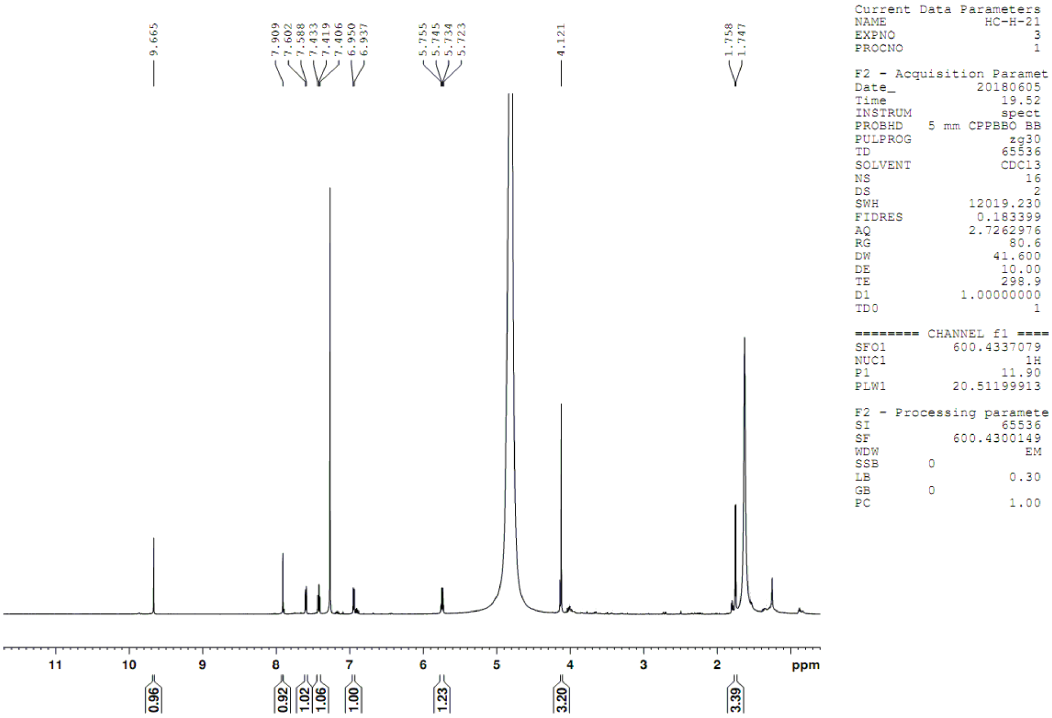

Figure S10. ^{1}H-NMR (600 MHz, $CDCl_3$) spectrum of the new compound **3**

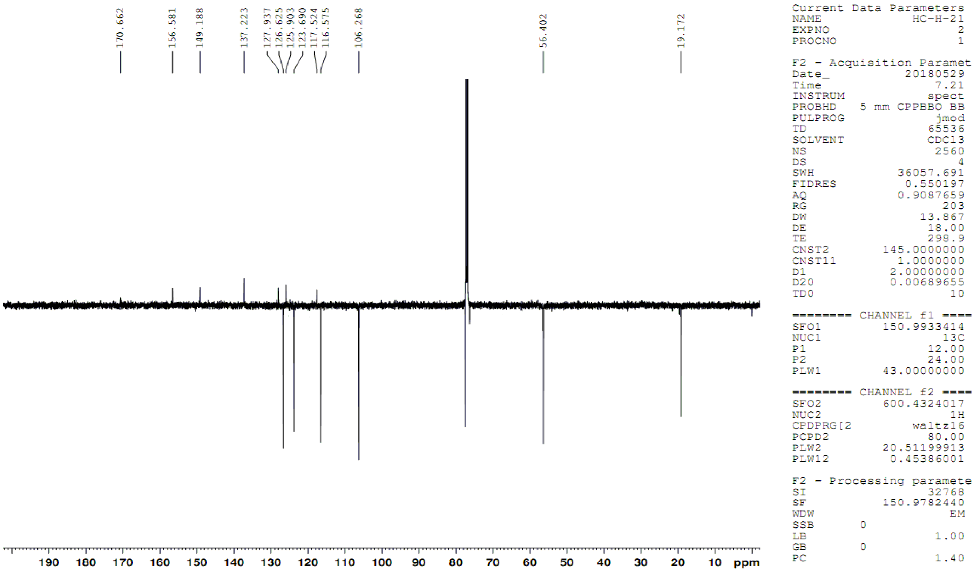

Figure S11. ^{13}C-APT (150 MHz, $CDCl_3$) spectrum of the new compound **3**

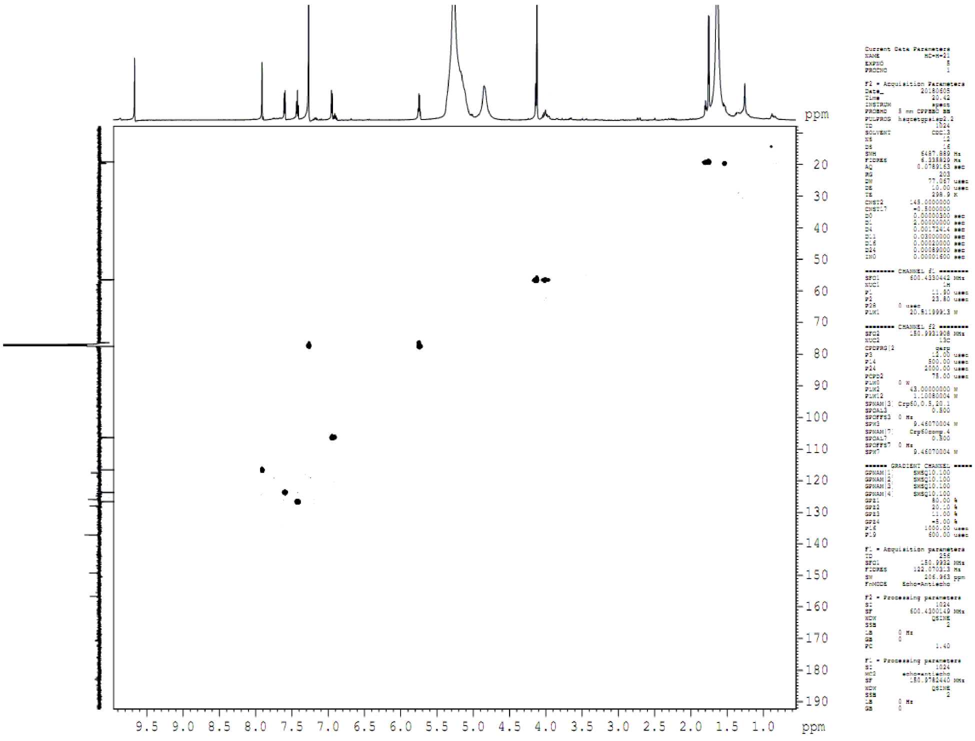

Figure S12. HSQC spectrum of the new compound **3**

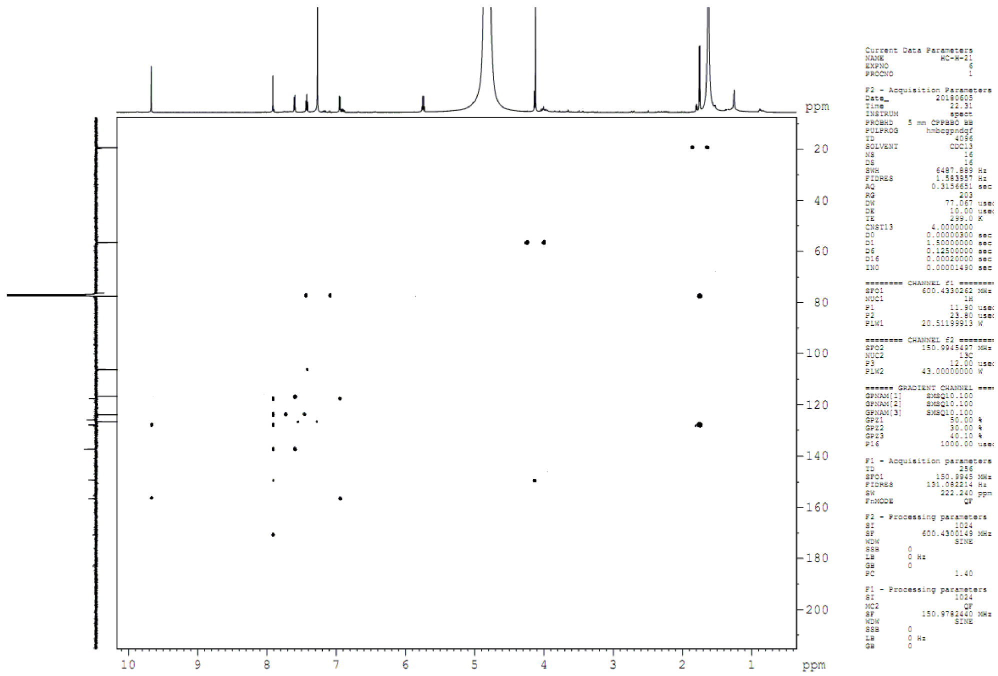

Figure S13. HMBC spectrum of the new compound **3**

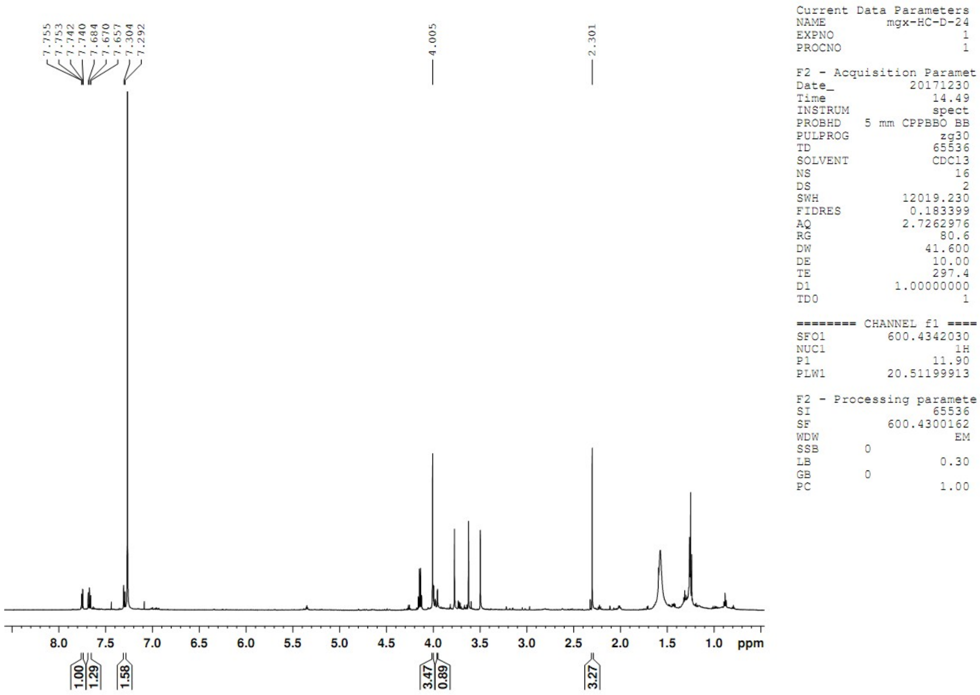

Figure S14. ^{1}H-NMR (600 MHz, $CDCl_3$) spectrum of the new compound **4**

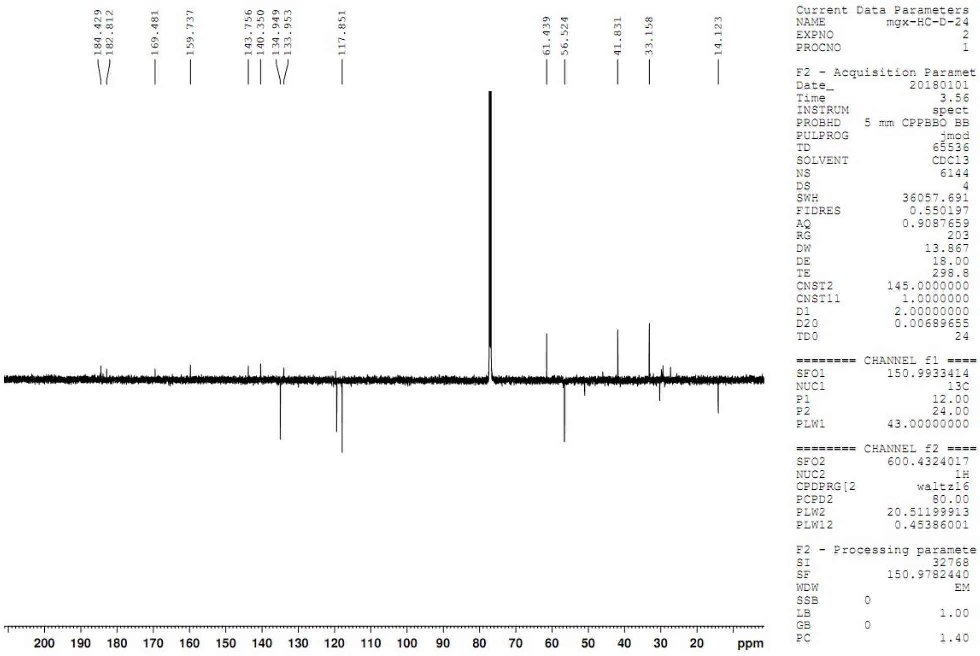

Figure S15. ^{13}C-APT (150 MHz, $CDCl_3$) spectrum of the new compound **4**

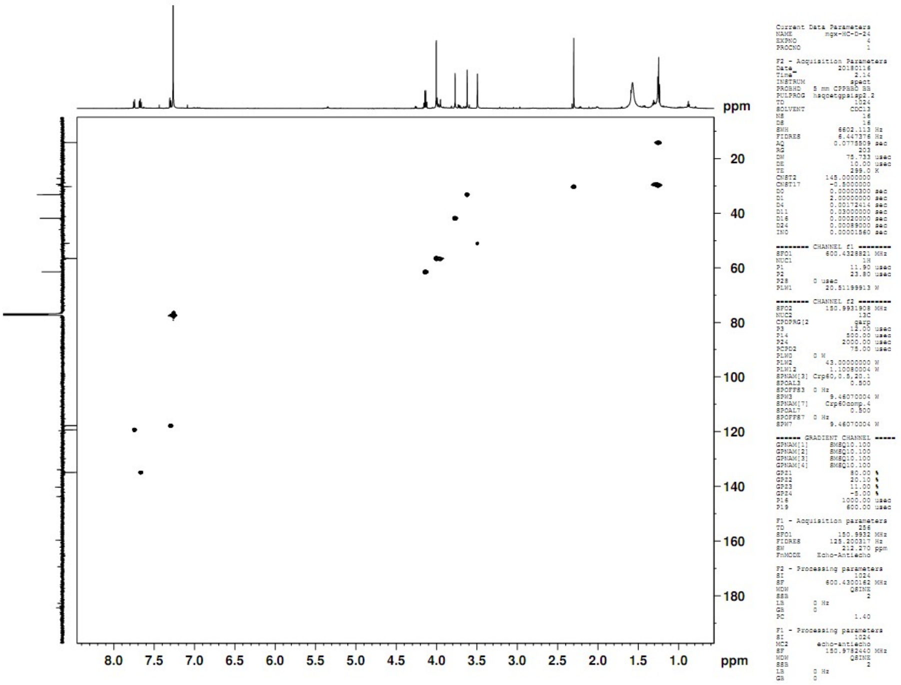

Figure S16. HSQC spectrum of the new compound **4**

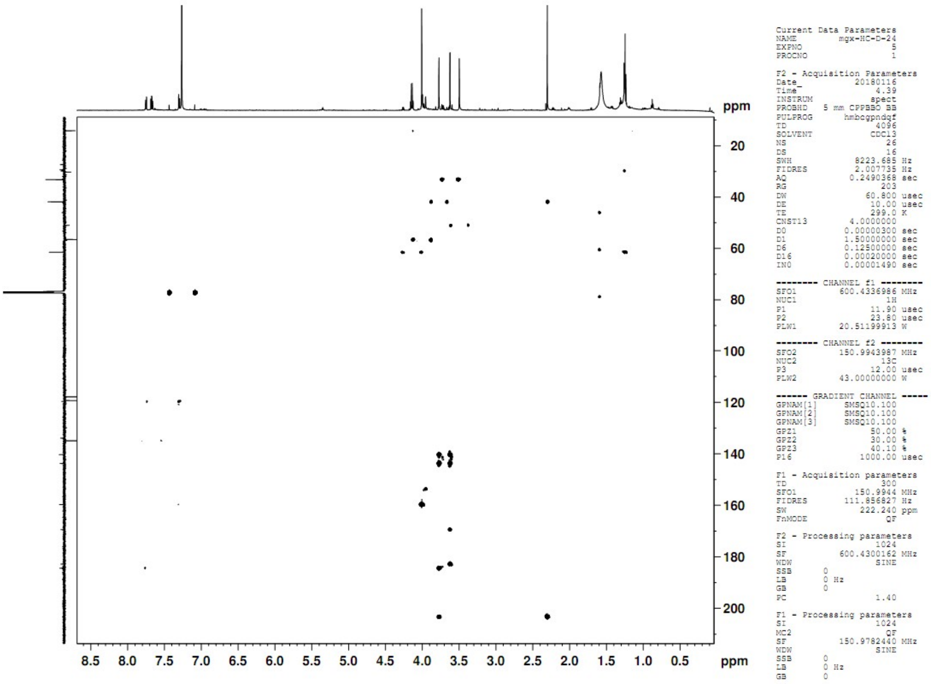

Figure S17. HMBC spectrum of the new compound **4**

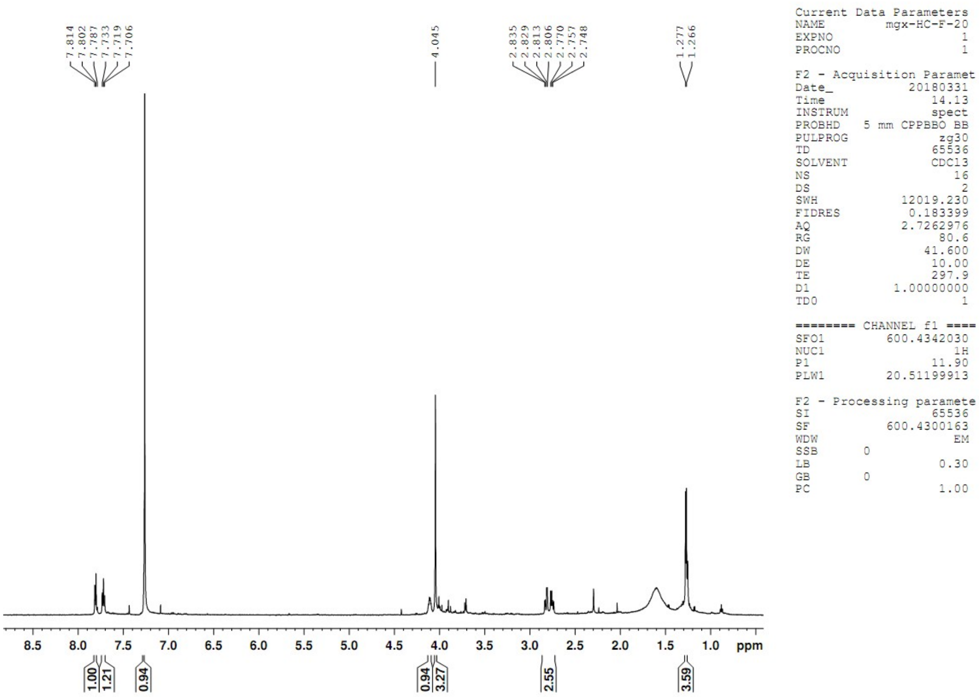

Figure S18. ^{1}H-NMR (600 MHz, $CDCl_3$) spectrum of the new compound **5**

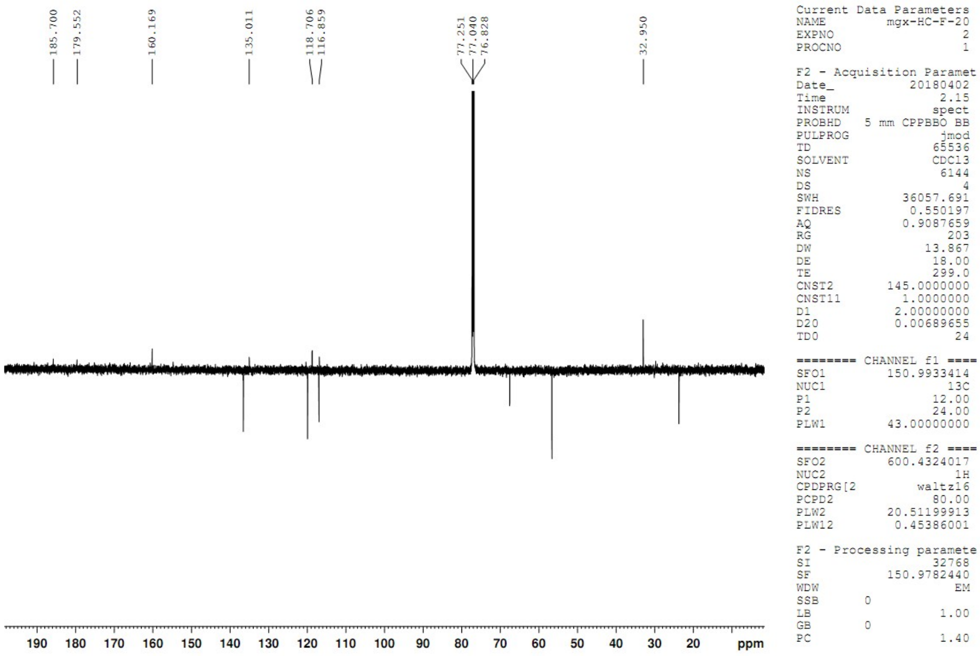

Figure S19. ^{13}C-APT (150 MHz, $CDCl_3$) spectrum of the new compound **5**

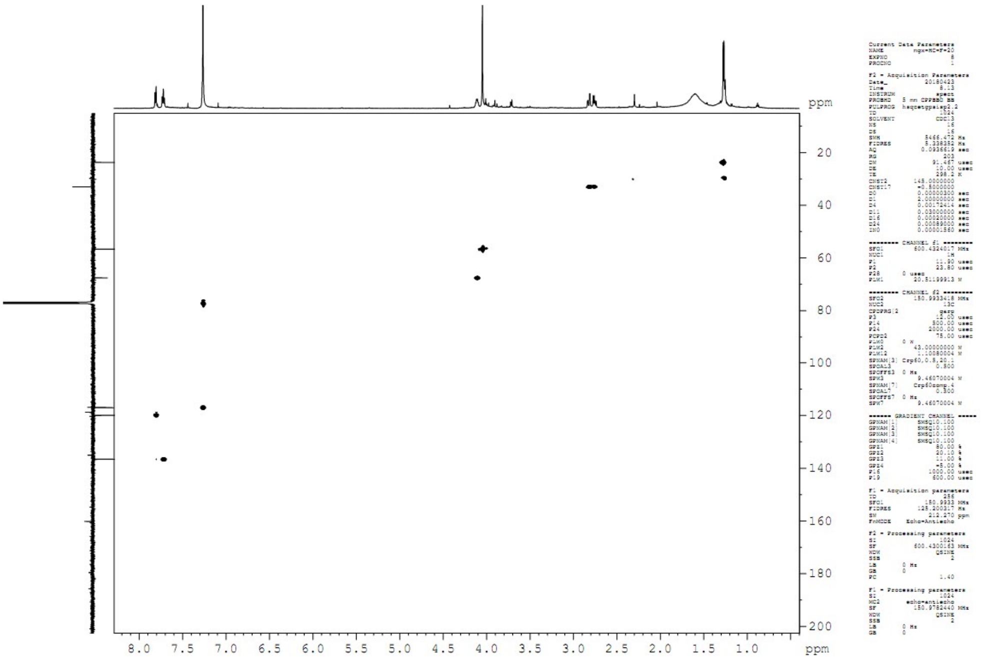

Figure S20. HSQC spectrum of the new compound **5**

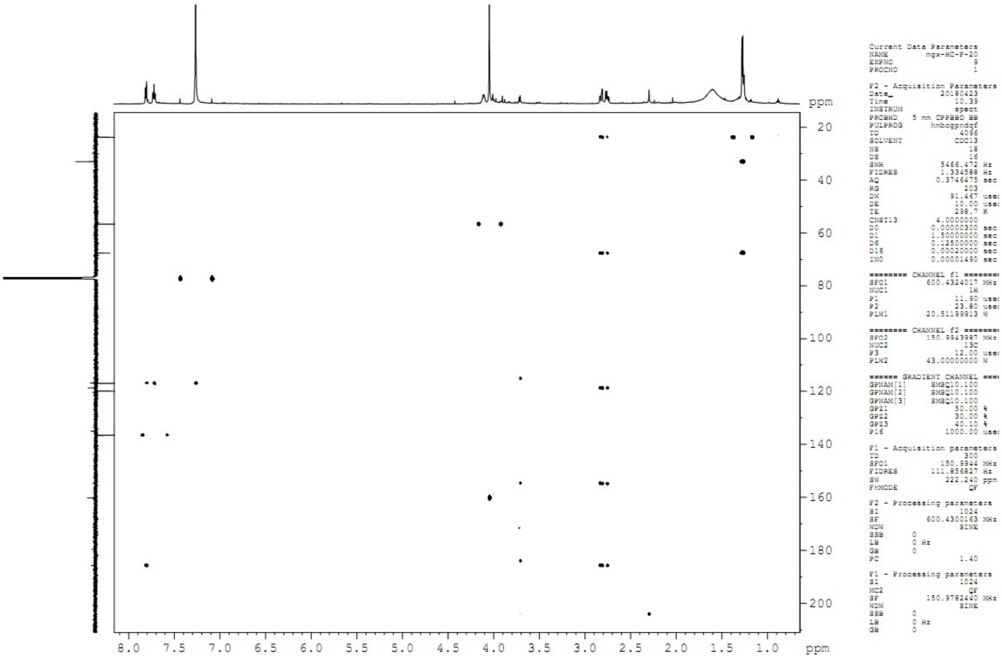

Figure S21. HMBC spectrum of the new compound **5**

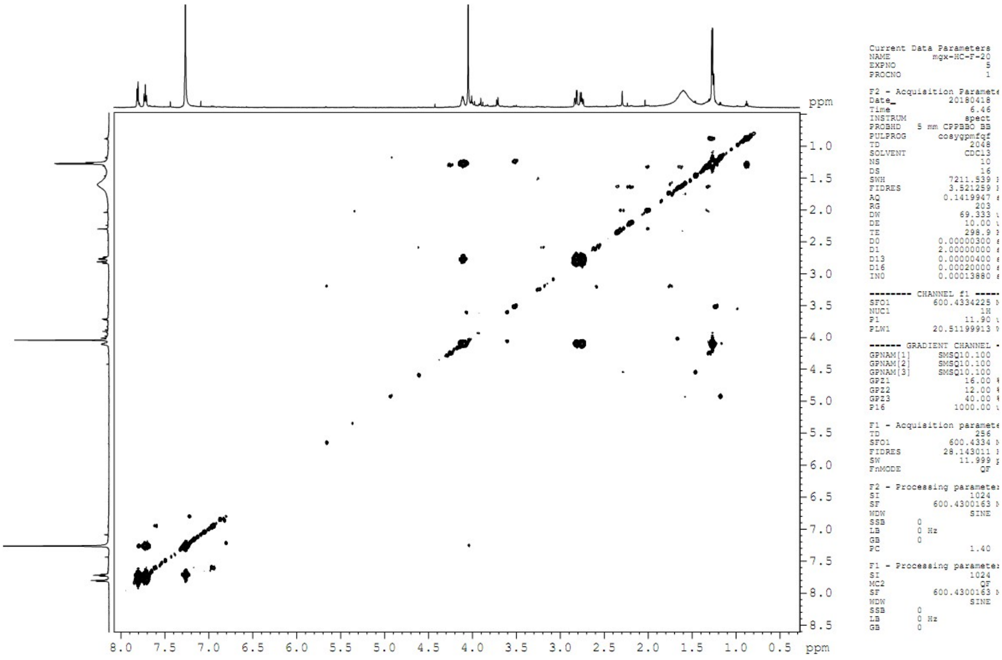

Figure S22. ^{1}H-^{1}H COSY spectrum of the new compound **5**

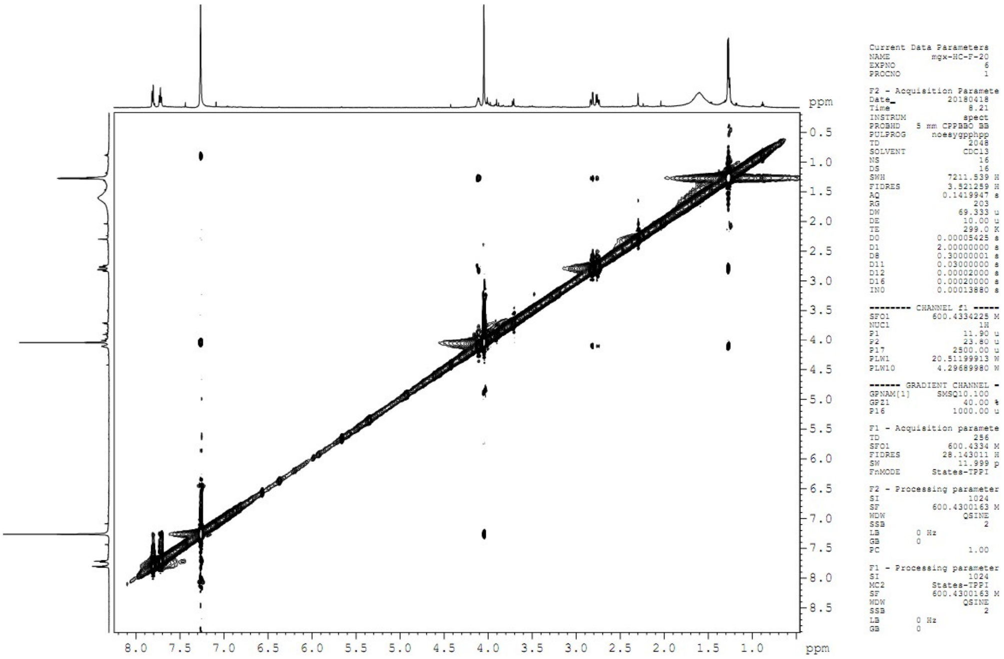

Figure S23. NOESY spectrum of the new compound **5**

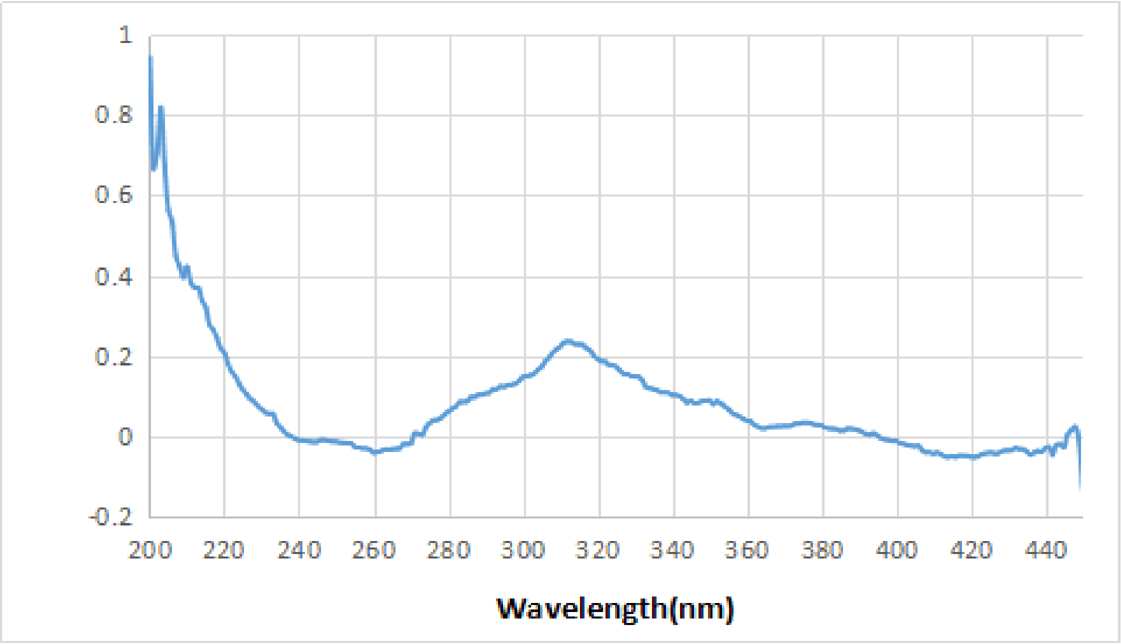

Figure S24. ECD spectrum of the new compound **1** in MeOH

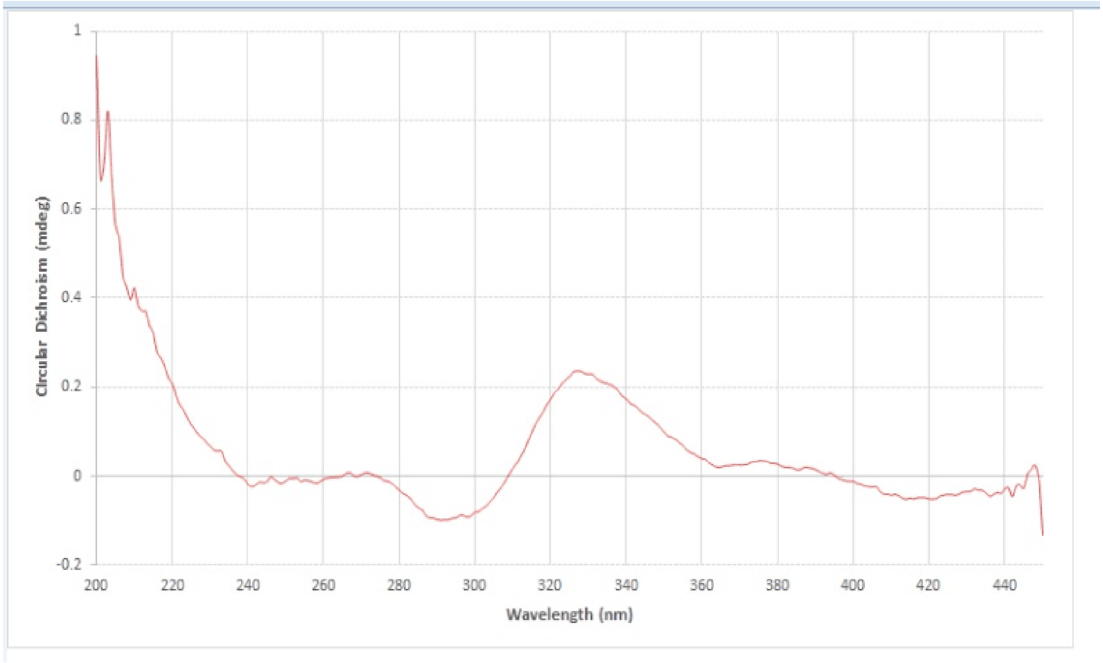

Figure S25. ECD spectrum of the new compound **2** in MeOH

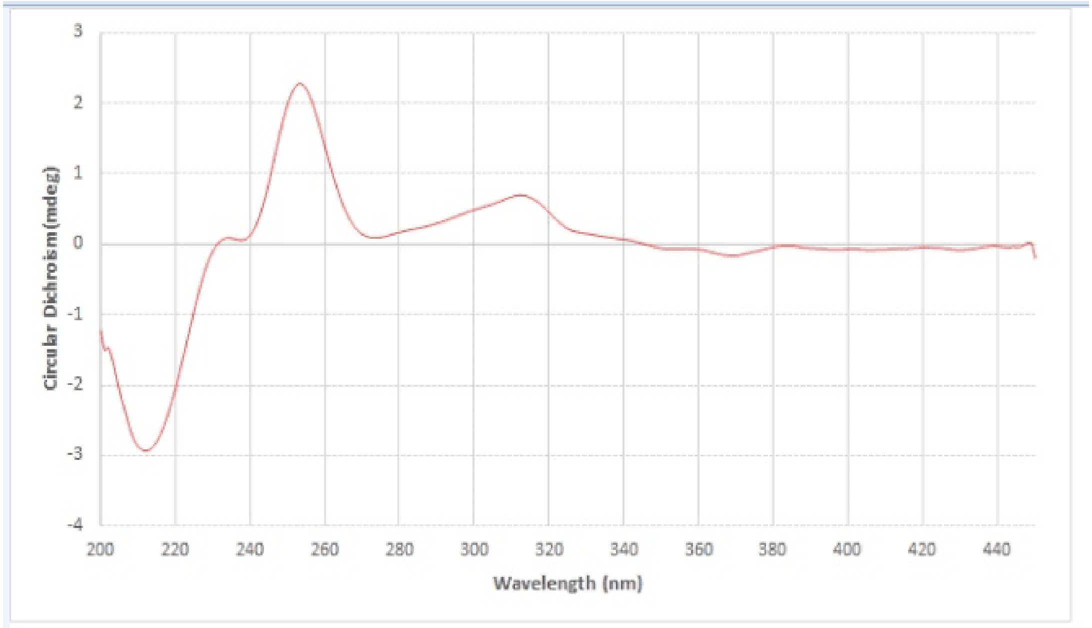

Figure S26. ECD spectrum of the new compound **3** in MeOH
